# Daily Supplementation of High Doses of Tocotrienol-Rich Fraction From Palm Oil Produced No Toxic Effects in Healthy Mice

**DOI:** 10.1155/jt/9464952

**Published:** 2025-07-29

**Authors:** Nevvin Raaj Morgan, Saatheeyavaane Bhuvanendran, Purushotham Krishnappa, Ammu Kutty Radhakrishnan

**Affiliations:** ^1^Food as Medicine Research Strength, Jeffrey Cheah School of Medicine and Health Sciences, Monash University Malaysia, Bandar Sunway, Subang Jaya 47500, Selangor Darul Ehsan, Malaysia; ^2^Pathology Division, School of Medicine, IMU University, Bukit Jalil, Kuala Lumpur 57000, Malaysia

**Keywords:** sub-acute exposure, tocotrienol-rich fraction (TRF), toxicity, vitamin E

## Abstract

The vitamin E derived from palm oil, known as the tocotrienol-rich fraction (TRF), has been reported to possess potent anticancer and immunomodulatory effects in numerous cell-based and animal models of breast cancer (BC). However, only a low dose of TRF (50 mg/kg, equivalent to 1 mg/day) has been tested, which resulted in incomplete effects in a mouse model of BC. In addition, there are no scientific data on the toxic effects of TRF on the internal organs. This study aimed to evaluate the effects of supplementing higher doses of TRF on biochemical parameters and histology of internal organs in female BALB/c mice. In brief, 30 female BALB/c mice were randomly assigned to one of the six study groups (five mice/group). The mice were fed daily with vehicle (control), 50, 100, 150, 200, or 250 mg/kg of TRF for 28 days by oral gavage. The results show that the subacute exposure of TRF showed no toxic effects in the animals from all the groups, as evaluated through some biochemical tests (alanine transaminase (ALT), alkaline phosphatase (ALP), creatine, and urea) and histology of the liver. In conclusion, female BALB/c mice fed daily with 250 mg/kg of TRF showed no signs of distress or adverse effects.

## 1. Introduction

Vitamin E is a broad term for a group of lipid-soluble vitamins with a chromanol ring and a carbon side chain attached to it at position C2 [1]. There are two main groups of vitamin E, which are tocopherol and tocotrienols, that can further be divided into four different naturally occurring isoforms, namely, alpha (α), beta (β), gamma (γ), and delta (δ). Vitamin E, especially the alpha-tocopherol (αToc), is well known for its antioxidant activity. However, there is an increasing interest in tocotrienols (T3s) due to their enhanced antioxidant activity, which is attributed to their unsaturated isoprenoid side chain that makes them more permeable to tissue layers relative to their tocopherol counterpart that has a saturated phytyl side chain [2]. Moreover, studies have shown other unique properties of T3s, such as hypercholesterolaemic, neuroprotective, and anticancer effects [3, 4]. Some sources of T3 include annatto seed oil, rice bran oil, and palm oil [5]. The vitamin E extract of palm oil is called tocotrienol-rich fraction (TRF), which comprises about 30% αToc and 70% T3s [6]. Supplementation of 50 mg/kg of TRF daily has shown reduced tumor size, reduced rate of metastasis, and a higher population of cytotoxic T cells as well as natural killer (NK) cells in a syngeneic mouse model of breast cancer (BC) [7, 8]. Although the results are promising, the anticancer effects observed were not complete, which warrants evaluation of a higher dose of TRF supplementation in this mouse BC model. The higher dose of TRF has been used in other disease models, such as the sciatic nerve injury rat model (200 mg/kg TRF) [9] and the Alzheimer's disease model of the mouse (200 mg/kg TRF) [9, 10]. Moreover, in a phase II clinical trial with 23 patients, it was reported that supplementation with a higher than the conventional dose of δT3 showed promising anticancer effects in ovarian cancer patients [11]. Hence, the usage of high-dose TRF as an anticancer and immunotherapeutic agent is hypothesized to be beneficial in the mouse BC model. Although the high dose of T3s is shown to be beneficial as a therapeutic agent in various diseases, there are limited reports on the toxicity of orally supplemented low and high-dose T3s and their systemic effects remain unknown. Thus, this study aims to determine the subacute effects of orally supplemented dose-derived TRF as an ongoing effort to study the use of TRF as an anticancer agent in a syngeneic mouse model of breast cancer.

## 2. Methodology

### 2.1. Treatment Preparation

The TRF from palm oil was obtained from DavosLife E3. An appropriate amount of TRF was weighed and dissolved in medium-chain triglyceride (MCT) oil (Mymyracle Pte Ltd, Malaysia) to prepare the TRF for daily supplementation.

### 2.2. Animal Experimentation

A total of 30 female BALB/c mice of 6 weeks of age were used in this experiment. The animals were housed in a mouse experiment room in the animal-holding facility at Monash University Malaysia. The study was conducted in accordance with the OECD guidelines, and the ethics approval for carrying out this study was obtained from the Monash University Animal Ethics Committee (Project ID: 37859). The mice were placed in plastic cages maintained at 25°C ± 2°C with a 12:12 dark/light cycle. The mice received *ad libitum* standard food pellets and water throughout the study. After a short acclimatization period, the mice were randomly assigned into one of the six study groups, namely, control group (*n* = 5), 50 mg/kg body weight (BW) TRF-treated group (*n* = 5), 100 mg/kg BW TRF-treated group (*n* = 5), 150 mg/kg BW TRF-treated group (*n* = 5), 200 mg/kg BW TRF-treated group (*n* = 5), and 250 mg/kg BW TRF-treated group (*n* = 5). The mice were fed daily with 50 μL vehicle (control) or varying doses of TRF in vehicle by oral gavage for 28 days. Upon euthanasia on Day 29, the blood and liver samples from the mice were collected for biochemical analysis and histopathological studies.

### 2.3. Animal Monitoring

Throughout the 28 days, the posture and mobility, activity level, fur conditions, behavior, and mortality of the mice were observed, and their BWs were measured once every 7 days using a benchtop electronic balance.

### 2.4. Biochemical Assay

On Day 29, the mice were anaesthetized using ketamine–xylazine (100 mg/kg:10 mg/kg), and blood was drawn from the heart via cardiac puncture. Blood samples were collected in plain tubes. The blood samples were centrifuged (5000 rpm for 10 min at 4°C) to separate the serum from the cellular components. The serum samples were transferred to 1.5 mL tubes and stored at −20°C until analysis. The levels of serum alanine transferase (ALT), alkaline phosphatase (ALP), creatinine, and urea were measured using an automatic biochemical analyzer (BA400).

### 2.5. Histopathological Samples

At autopsy, the liver was harvested from all the test animals and fixed in 10% buffered formalin for 48 h before the tissue was processed and embedded in molten paraffin. The tissues were cut into smaller sections (4 μm) using a microtome. The sections were stained with hematoxylin and eosin (H&E) and subjected to p-xylene-bis-pyridinium bromide mounting. The liver sections were analyzed using a light microscope to evaluate various features related to inflammation, necrosis, hepatic cell vacuolization, sinusoidal dilatation, and other abnormalities by a histopathologist blinded to the study. The pathology identified was further classified into mild, moderate, and severe, which are allotted a score (Table 1) according to the system developed by Knodell in 1981 [12, 13].

### 2.6. Relative Organ Weight (ROW) of Liver

The ROW of the liver was calculated using the following formula:

(1) $$\begin{array}{c} \text{ROW}=\frac{\text{Liver weight}}{\text{BW}}\times 100. \end{array}$$

### 2.7. Statistical Analysis

All data are expressed as the mean, standard error of the mean (SEM). Statistical evaluation of the data was done using one-way analysis of variance (ANOVA) followed by Tukey post hoc tests in GraphPad Prism Version 10.2.2 (341). *p* < 0.05 is considered significant.

## 3. Results

### 3.1. Clinical Observation and BW Measurement

**Figure 1:**
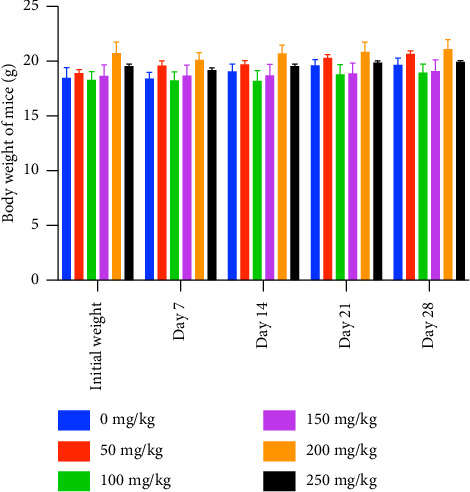
Effect of TRF on the body weight of mice. Values are expressed as the mean ± SEM (*n* = 5). There are no significant differences among groups (ANOVA and Tukey's test post hoc, *p* > 0.05). The data passed the Shapiro–Wilk test for normality.

The clinical observations and BWs of mice in the control and treatment groups are shown in Table 2. All the mice showed no signs of toxicity, no changes in behavior. In addition, there were no significant changes in BW and no mortality (Figure 1).

### 3.2. Biochemical Analysis

**Figure 2:**
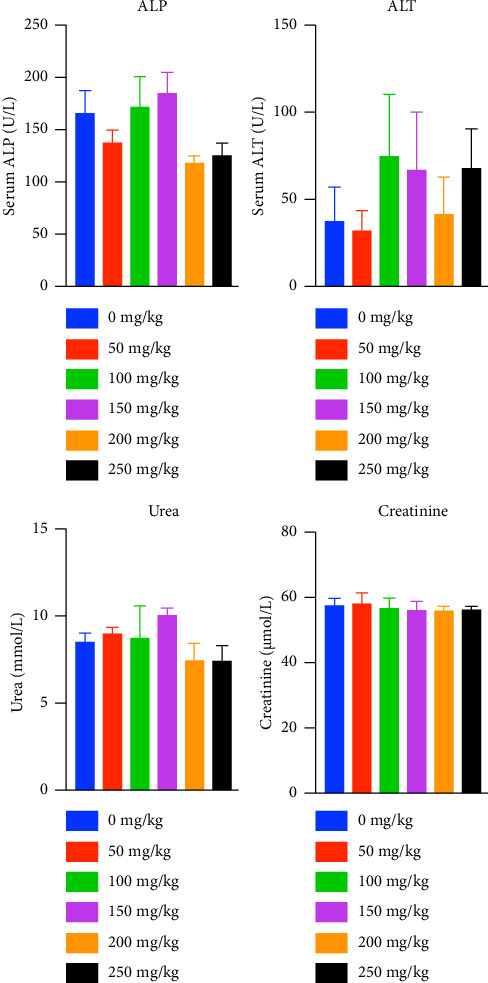
Effect of TRF on the biochemical parameters depicting liver and kidney functions. Values are expressed as the mean ± SEM (*n* = 3). There are no significant differences among groups (ANOVA and Tukey's test post hoc, *p* > 0.05). The data passed the Shapiro–Wilk test for normality.

Two serum markers, ALT and ALP, were quantified to assess any liver injury following TRF treatment. There was no significant increase observed in the serum ALT and ALP levels between the six experimental groups (Figure 2). In addition, there was no significant difference among the six study groups on the two biochemical tests used to check on kidney function, that is, urea and creatinine (Figure 2). These results suggest that daily supplementation of high doses up to 250 mg/kg of TRF for 28 days had no toxic effects on the BALB/c mice.

### 3.3. Liver Histopathology

**Figure 3:**
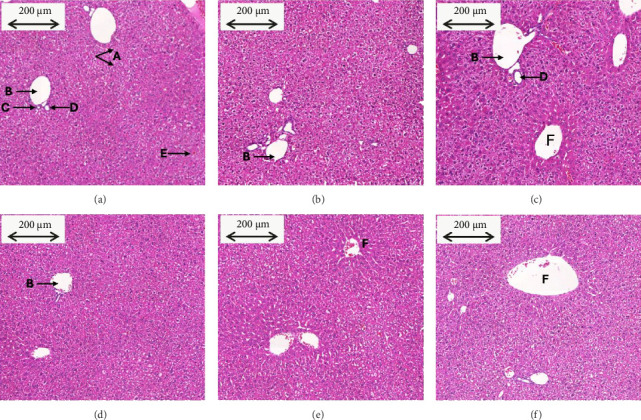
Photomicrographs of the liver histopathology sections (10X magnification) taken from 6 weeks old female BALB/c mice fed daily by oral gavage with (a) vehicle (MCT oil); (b) 1 mg TRF (50 mg/kg BW); (c) 2 mg TRF (100 mg/kg BW); (d) 3 mg TRF (150 mg/kg BW); (e) 4 mg TRF (200 mg/kg BW); or (f) 5 mg TRF (250 mg/kg BW). There were no signs of toxicity observed in any of the liver sections. This is a representative of the three samples analyzed (Note: A: hepatocytes showing mild vacuolization; B: branch of normal appearing portal vein; C: branch of normal appearing bile duct; D: branch of normal appearing hepatic artery; E: sinusoids showing mild congestion; F: central vein; BW: body weight; MCT: midchain triglyceride; TRF: tocotrienol-rich fraction).

Photomicrographs of liver sections from all the groups showed mildly edematous liver parenchyma with intact architecture, hepatocyte vacuolization, and minimal inflammation of periportal areas (Figure 3). Some sections showed focal areas of necrosis. These changes were seen in varying severity among the different animal groups and within the same animal groups. However, the variation in the severity of these findings was not significant. The severity score for liver histopathological changes, such as the presence of inflammation, necrosis, hepatocyte vacuolization, and sinusoidal dilation, is tabulated in Table 3. Overall, the severity score shows no clinical significance in the pathological changes of the liver sections across the different groups.

### 3.4. ROW of Liver

**Figure 4:**
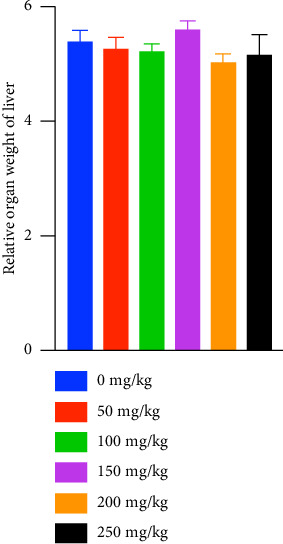
Effect of daily supplementation of various doses of TRF for 28 days on the relative organ weight (ROW) of livers of mice from the six study groups. The values are expressed as the mean ± SEM (*n* = 3). There are no significant differences among groups (ANOVA and Tukey's test post hoc, *p* > 0.05). The data passed the Shapiro–Wilk test for normality.

There are no significant changes in the ROW of livers taken from the mice from the six study groups (Figure 4).

## 4. Discussion

A 28-day oral toxicity test for evaluating the effects of compounds in organ systems is regarded as a standard and fundamental safety assessment [14, 15]. Although previously, the in vitro results have shown that TRF has cytotoxic effects on cancer cells (4T cell line) but not on normal cells (mouse dendritic cells), it is vital to conduct a toxicity test in vivo to study the safety of different doses of TRF in the whole organism and their major organs, such as liver and kidneys [7].

Changes in BW are one of the important indicators of acute toxicity of chemicals or drugs in small animals such as BALB/c mice [16]. In the present study, daily oral supplementation of TRF for 28 days showed no statistically significant changes in the mice's BWs. Moreover, the mice in all the groups survived beyond the 28 days of observation with no signs of distress or toxicity. In addition, the feeding rate, water intake, and the feces of the mice in all the groups were normal during this subacute study. Since there was no mortality observed even in the high-dose treatment group, it is safe to say that the 50% lethal dose (LD50) of TRF in the six-week-old female BALB/c mice exceeds 250 mg/kg BW [17].

The liver is one of the significant organs known for its role in detoxification, metabolism, as well as biotransformation and biodistribution of xenobiotics [18]. Hepatotoxicity is a condition in which the liver is exposed to injury caused by foreign agents, such as phytochemicals, due to the liver's function in drug metabolism [19]. Quantifying serum enzymes to determine hepatocellular injury is crucial in diagnosing chronic liver diseases or dysfunctions [20]. ALT is an enzyme found mainly in the liver, and marked elevation in its levels indicates inflammation and toxic liver injury [21]. In the present study, we have shown that even at high doses of TRF supplementation, there is no significant difference in the levels of serum ALT, and their readings are within the normal limit, indicating normal liver function [22]. Although the function of AST is similar to ALT, which is to catabolize amino acids, ALT is chosen as a more sensitive marker for liver injury, as ALT is primarily found in the liver. In contrast, AST can be found in other tissues such as the heart, pancreas, lungs, and brain [21, 23]. On the other hand, ALP is also one of the enzymes tested to evaluate the liver's normal function, as its elevation in serum suggests damage in the epithelial cells of the canalicular membrane or biliary ducts [24]. The ALP levels of the treated and the control groups were within the normal range, and there was no significant difference between the groups, eliminating the presence of drug-induced cholestasis. The pathological changes in the liver sections of various groups are comparable with minimal variations. The cellular changes like vacuolization were seen in mild form from the liver sections across all the animal groups of this study. The inflammatory cells are sparsely seen in many liver sections of different study animal groups without any specific patterns. Very few sections also showed patchy focal areas of necrosis in some of the animal groups of the study. The identified pathological findings are mild and random across the study groups. This suggests that findings are clinically insignificant. Most of these changes could have been produced by other factors, such as laboratory techniques such as sample procurement and fixation. Moreover, there are no significant differences between the ROW of livers between the groups, indicating no signs of injury, atrophy, or tumidity in the livers of the mice [15]. Besides the liver, the kidneys are also one of the important target organs in toxicity studies due to their role as the major excretory organ. End products of amino acid metabolism, creatinine and urea, are chosen as biochemical parameters to check for nephrotoxicity. Elevation of their levels above the normal range may indicate the kidneys' failure to remove them, possibly caused by injuries. The results of this study show that upon subacute supplementation of different doses of TRF, there is no significant difference in the levels of creatinine and urea, and it is notable that their levels are found to be within the normal range, indicative of normal glomerular filtration rate and kidney functions [22, 25]. These results are also similar to a study that showed no adverse effects on the tissues or organs of CD2F1 mice when up to 300 mg/kg of gamma and delta T3 was administered subcutaneously [26]. Although the current findings ensure the safety of TRF on major organs such as the kidney and liver, due to the lack of resources, the toxicity of TRF in other systems, such as the reproductive and nervous systems, was not evaluated. Moreover, this study was done only using female BALB/c mice; data involving male BALB/c mice are unavailable. Taking everything into account, oral supplementation of 5 mg (250 mg/kg) of TRF is considered safe, and its anticancer immune effects in a syngeneic mouse model of breast cancer can be evaluated in future.

## 5. Conclusion

Oral administration of up to 5 mg (250 mg/kg BW) of TRF for 28 days was well tolerated in female BALB/c mice and exhibited no adverse effects or mortality. The subacute exposure demonstrated no changes in the behavior, biochemical parameters evaluating renal and hepatic functions, and histopathological findings, confirming that TRF supplementation up to 250 mg/kg BW is nontoxic. Future studies studying the effects of TRF in male BALB/c are needed alongside studies involving the long-term effects of high-dose TRF supplementation to corroborate the current results for the translation to human consumption. Since the highest dose (250 mg/kg BW) of TRF has been shown to be well tolerated in healthy female BALB/c mice, their anticancer effects in the syngeneic mouse breast cancer model can be further evaluated in the future to elucidate the mechanisms of TRF in eliciting anticancer immune responses.

## Figures and Tables

**Table 1:** Severity scores of the histopathology changes.

Score	Description
1	Mild
2	Moderate
3	Severe

**Table 2:** Health monitoring and clinical observations of mice.

TRF dose in vehicle (MCT oil)	Posture and mobility	Alertness and activity	Eyes	Nose	Coat and skin	Breathing	Faeces	Food and water intake
0 mg/kg TRF	Normal	Normal	Clear and moist	Open and clear	Sleek and shiny	Normal	Normal, no signs of diarrhea	Normal
50 mg/kg BW TRF	Normal	Normal	Clear and moist	Open and clear	Sleek and shiny	Normal	Normal, no signs of diarrhea	Normal
100 mg/kg BW TRF	Normal	Normal	Clear and moist	Open and clear	Sleek and shiny	Normal	Normal, no signs of diarrhea	Normal
150 mg/kg BW TRF	Normal	Normal	Clear and moist	Open and clear	Sleek and shiny	Normal	Normal, no signs of diarrhea	Normal
200 mg/kg BW TRF	Normal	Normal	Clear and moist	Open and clear	Sleek and shiny	Normal	Normal, no signs of diarrhea	Normal
250 mg/kg BW TRF	Normal	Normal	Clear and moist	Open and clear	Sleek and shiny	Normal	Normal, no signs of diarrhea	Normal

Abbreviations: BW, body weight; MCT, midchain triglyceride; TRF, tocotrienol-rich fraction.

**Table 3:** Severity score for changes observed in the liver histopathological sections.

Dose of TRF in vehicle (MCT oil) fed daily	Score (*n* = 3 mice/group)			
	Inflammation	Necrosis	Hepatocyte vacuolization	Sinusoidal dilation
0 mg/kg BW TRF	0 (*n* = 2)	0 (*n* = 3)	0 (*n* = 1)	0 (*n* = 3)
	1 (*n* = 1)		1 (*n* = 1)	
			2 (*n* = 1)	

50 mg/kg BW TRF	0 (*n* = 2)	0 (*n* = 3)	0 (*n* = 1)	0 (*n* = 3)
			1 (*n* = 2)	
	1 (*n* = 1)			

100 mg/kg BW TRF	0 (*n* = 3)	0 (*n* = 3)	1 (*n* = 3)	0 (*n* = 3)

150 mg/kg BW TRF	0 (*n* = 2)	0 (*n* = 1)	1 (*n* = 1)	0 (*n* = 3)
	1 (*n* = 1)	1 (*n* = 2)	2 (*n* = 2)	

200 mg/kg BW TRF	0 (*n* = 3)	0 (*n* = 3)	0 (*n* = 2)	0 (*n* = 3)
			1 (*n* = 1)	

250 mg/kg BW TRF	0 (*n* = 1)	0 (*n* = 3)	1 (*n* = 2)	0 (*n* = 2)
	1 (*n* = 2)		2 (*n* = 1)	1 (*n* = 1)

*Note: n*: Number of mice.

Abbreviations: BW, body weight; MCT, midchain triglyceride; TRF, tocotrienol-rich fraction.

## Data Availability

The data that support the findings of this study are available from the corresponding author upon reasonable request.
